# The social origins of obesity within and across generations

**DOI:** 10.1111/obr.13514

**Published:** 2022-11-02

**Authors:** Erik Hemmingsson, Paulina Nowicka, Stanley Ulijaszek, Thorkild I. A. Sørensen

**Affiliations:** ^1^ Department of Physical Activity and Health The Swedish School of Sport and Health Sciences Stockholm Sweden; ^2^ Department of Food Studies, Nutrition, and Dietetics Uppsala University Uppsala Sweden; ^3^ Unit for Biocultural Variation and Obesity, School of Anthropology and Museum Ethnography University of Oxford Oxford UK; ^4^ Department of Public Health and Novo Nordisk Foundation Center for Basic Metabolic Research, Faculty of Health and Medical Sciences University of Copenhagen Copenhagen Denmark

**Keywords:** chronic stress, genetics, social adversity, social transmission, weight stigma

## Abstract

We propose a model for obesity development that traces a considerable part of its origins to the social domain (mainly different forms of prolonged social adversity), both within and across generations, working in tandem with a genetic predisposition. To facilitate overview of social pathways, we place particular focus on three areas that form a cascading sequence: (A) social adversity within the family (parents having a low education, a low social position, poverty and financial insecurity; offspring being exposed to gestational stress, unmet social and emotional needs, abuse, maltreatment and other negative life events, social deprivation and relationship discord); (B) increasing levels of insecurity, negative emotions, chronic stress, and a disruption of energy homeostasis; and (C) weight gain and obesity, eliciting further social stress and weight stigma in both generations. Social adversity, when combined with genetic predisposition, thereby substantially contributes to highly effective transmission of obesity from parents to offspring, as well as to obesity development within current generations. Prevention efforts may benefit from mitigating multiple types of social adversity in individuals, families, and communities, notably poverty and financial strain, and by improving education levels.

## 1 INTRODUCTION

There has been little progress in the prevention of obesity through conventional diet and exercise programs,^1^ which are primarily founded on a simplified causal interpretation of excess calorie intake. Mounting evidence suggests that various psychosocial aspects, including social adversities, may play a more profound role in obesity development than by just being associated with excess calorie intake induced by abundance of food in the so‐called obesogenic environments.

Given that human life is fundamentally performed in a social context, it is entirely plausible that social factors influence the development of obesity, provided there is sufficient dietary energy in the surrounding environment to satisfy the energy needs in the concomitantly growing, metabolically active lean body mass (an energy requirement that far exceeds the minute increment of energy stored in the fat tissue).^2^ Indeed, the close link between social factors such as poverty and inequality with obesity has been documented at least since the mid‐1960s,^3^ notably first by the work of Albert J. Stunkard and coworkers.^4^, ^5^

In this paper, we argue that excessive body fatness may be due to prolonged exposure to social adversity,^6^ especially if social challenges are present early in life, when cell biology is at peak malleability.^7^ Indeed, evidence has accumulated over many decades linking different types of social adversity, such as poverty, low socioeconomic status, neglect, maltreatment, and abuse with higher rates of obesity,^3^, ^8^, ^9^, ^10^ especially in market liberal countries.^11^

## 2 AIMS AND RATIONALE

The overall aim is to provide an integrating model of social factors, predominantly relating to adversity, in the origins (here defined as early, or upstream, influences on psychological, emotional, and biological processes that promote a growing adipose mass) of obesity, co‐acting with genetic influences. A secondary aim, conditional upon the first, is to identify clear opportunities for opposing social exposures of relevance to obesity prevention.

Although there is a considerable body of theoretical and synthesizing literature that can help to frame the impact of social adversity on health and body weight, for example, within the fields of developmental psychology, social ecology, and behavior,^12^, ^13^, ^14^, ^15^, ^16^, ^17^, ^18^, ^19^, ^20^, ^21^, ^22^ we have primarily based our selection of factors on the available empirical evidence from studies of the association of such adversities with obesity development, such as neglect, maltreatment, abuse, and weight stigma, but also other psychosocially challenging or traumatic life events.^8^, ^23^, ^24^, ^25^, ^26^, ^27^

Although our model encompasses all life stages, we place particular emphasis on childhood. The rationale for doing this is threefold: Firstly, children with increased body weight are at increased risk of obesity later in life.^28^ Secondly, adipocyte quantity appears to be largely determined during childhood.^29^ Thirdly, we wish to explore the phenomenon of how obesity in parents transmits to their offspring with a very high degree of probability.^30^, ^31^, ^32^ Finally, it must be emphasized that we consider this model to be applicable only under food abundant conditions, because obesity development is much less likely during conditions of food scarcity for the reasons mentioned above.

## 3 THE INTEGRATING MODEL

### 3.1 Genetic and environmental influences

**FIGURE 1:**
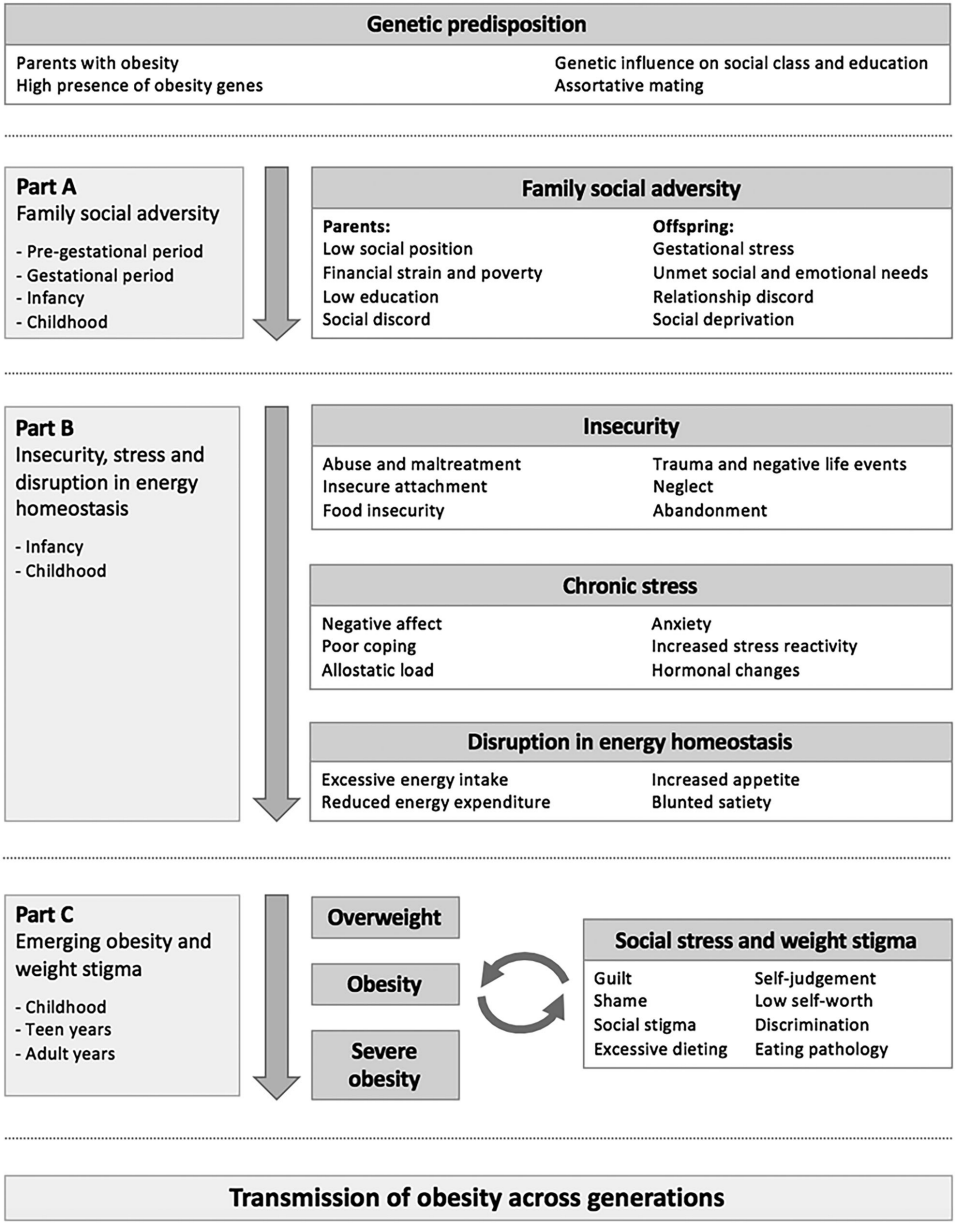
Overview of the integrating model of the social origins of obesity through a combination of genetic predisposition with social adversity in both parents and their offspring, which cascade towards the promotion of insecurity, chronic stress, weight gain and obesity, and finally weight stigma. While the model is presented as being approximately linear, that is, cascading from Part A → Part B → Part C, there are likely to be several (and potentially synergistic) co‐acting feedback mechanisms involved (circular causality processes), which will require much additional scientific scrutiny to establish (or disprove). Social adversity pathways to obesity apply both to a potent intergenerational transmission from parents to offspring (Part A only) and to obesity development within current generations (some of Part A [offspring] and all of Parts B and C).

It has been known for over a century that obesity “runs in families.” An integrating model of obesity (Figure 1) must necessarily therefore include familial influences.^33^, ^34^ Generally, the body mass index (BMI) as a continuous trait exhibits familial correlation, which means that when investigating numerous families within a population, the BMI of one member of a nuclear family, constituted by parents and their offspring, correlates with the BMI of the other family members.

Thus, there is consistent evidence showing that obesity in the parents substantially increases the risk of offspring obesity,^30^, ^31^, ^32^ a key factor in cross‐generational transmission. This includes a clear graded increase in the risk of childhood obesity depending on parental weight status, with much increased risk if both parents had developed obesity (compared with normal weight and overweight parents) and, in particular, severe obesity.^31^ Part of this effect, especially for children with severe obesity, might be attributed to assortative mating behavior (phenotypic assortment).^35^, ^36^ There is some indication that the intergenerational transmission effect may be more pronounced in girls than boys, although still very strong in boys.^37^

The origin of such familial trait correlations is due to the common genetic and/or shared environmental influences on the trait. During the last several decades, multiple studies have aimed at disentangling the role of genes transmitted among biological family members from the role of the environment shared by family members in creating familial similarities in BMI and obesity. The main approach in resolving this issue has been the application of adoption and twin studies that complement each other by resting on different assumptions. Resemblance in BMI between adopted‐away individuals and BMI of their biological family members likely expresses genetic influence, and resemblance in BMI with adoptive family members reflects the influence of shared environment. In twin studies, the difference in resemblance of BMI between monozygotic and dizygotic twin pairs is considered to be due to genetic effects, whereas what remains of resemblance between twin pairs is considered to be due to their shared environment. Both methods also provide estimates of the contribution of the non‐shared environment to differences between individuals in the same population.

The key message emerging from a small number of adoption studies and many twin studies is that resemblance in BMI of adult biological family members no longer sharing a common household environment is almost entirely attributable to genetic influences.^34^, ^38^, ^39^, ^40^ However, shared environment does have some, though weaker, influence when family members are living together in the same household.^33^, ^34^, ^41^, ^42^, ^43^, ^44^, ^45^ This is in contrast to the prevailing expectation that habits are adopted in the shared environment and are presumed to be responsible for the effects that are carried through to later life outside the family environment.

Given this pattern, the question is whether genetic predisposition, as assessed by family relationships of adoptees and of twins, is dependent on the social environment.^46^ A twin study on the influence of education on BMI has shown a slight weakening of the effects of individual genetic differences by higher education in women, but not among men.^47^ A larger multi‐cohort twin study indicates that higher parental education weakly suppresses such genetic effects.^48^

One adoption study has shown that the social position of adoptive parents, assessed by ranking their occupation, maintains an influence on the BMI of offspring in adulthood, although there is no relation to the BMI of the adoptive parents.^49^ This finding suggests that parental social factors may have an influence on BMI, which operates independently of genetic transmission of predisposition to obesity.^49^ On the other hand, further analyses of this and another adoption study suggest that there are two pathways of effect of parental social factors on offspring BMI.^50^ The analyses confirm that there is an environmental effect of parental social factors operating irrespective of the BMI of the parents. However, the finding that social factors of the biological parents irrespective of their BMI remains associated with offspring BMI suggests that there is in addition a common genetic influence—a pleiotropic effect—on parental social factors and offspring BMI. In addition, genetic factors have been found to influence social factors such as education and social class, suggesting that genetic factors are (indirectly) involved in the promotion of social adversity.^49^, ^51^, ^52^, ^53^, ^54^, ^55^, ^56^ These complex relationships require replication and further investigation.

There is also evidence of a purely social “transmission” of obesity, that is, social exposures seemingly operating outside the genetic domain. This claim is based on findings that obesity tends to develop more in some social clusters than others,^57^ both inside and outside the family. Associations in BMI tend to be somewhat stronger for social ties outside the family, suggesting that this effect is not due to shared genetics. Indeed, the risk of developing obesity is much increased if a friend (57% increased risk) or spouse (37%) develops obesity,^57^ possibly through shared norms and other social ties.^58^ Social network analyses show adults and adolescents with overweight or obesity are more likely to have friends with overweight or obesity.^57^, ^59^ Both stigmatization by institutions and from those without obesity^23^ and mimicking of others with obesity^60^ are potential mechanisms whereby obesity can spread within a family or a social network.^61^ Given that such social transmission can take place at various stages of life, Warin et al^62^ have developed the framework of *biohabitus* for understanding how social and biological environments interact across the life course and may be transmitted and transformed across generations.

Although there has been much discussion of epigenetic influences on body weight,^63^ little progress has been made in identifying the specific epigenetic modulations by genomic methylation studies that relate to childhood obesity. Those found suggest that the observed methylation profile is rather an accompanying or resulting alteration.^64^, ^65^, ^66^, ^67^, ^68^, ^69^, ^70^

To summarize, both the individual genetic profile and the environment that the individual is exposed to strongly influence the development of BMI and obesity. Arguably, one of the strongest documented risk factors for childhood obesity is when both parents have obesity. The shared environment of a family plays a role in creating resemblances in BMI and obesity among family members as long as they live together, but it vanishes when they live apart. However, studies suggest that parental social factors may have lasting influence on offspring BMI both as purely environmental effect and through common genetic effects on social factors and BMI.

### 3.2 Part A: Family social adversity

Social family factors, broadly defined as repeated social interactions in the family environment, share several similarities to the transmission of a genetic predisposition from the biological parents: both are active from early in life and both have a strong influence on psychological, emotional and physiological factors pertinent to obesity development (Figure 1). Although a healthy and supportive family environment can play a protective role against obesity,^18^ there is ample evidence of the destructive influence of social adversity within the family.^8^, ^24^, ^25^

Some key social adversity indicators of the parent include being of low social class and low subjective social status, living under economic strain and poverty, living in a deprived area, and having low education status, all of which have been linked with obesity.^3^, ^71^ Additional aspects of low social class include uncertain employment, uncertain accommodation (immediate security, future security, discounting the future), effort–reward imbalance, and low social and cultural capital (education, occupational prestige, authority, community standing, knowledge, intellect),^72^ resulting in increased risk of parental chronic stress and psychological strain.^73^ Moreover, prolonged social adversity in adults also increases the likelihood of unhealthy behaviors such as smoking and excessive alcohol consumption, further contributing to a possible buildup of an insecure social environment when starting a family.

Prolonged social adversity is in turn associated with several debilitating psychological and emotional consequences in offspring: insecurity, chronic stress, and mental health problems, including depression, anxiety, negative belief systems, and negative affect (anger, apathy, hopelessness, frustration, distress, shame, guilt).^18^ There is increasing evidence that parental social adversity affects weight gain in the children.^74^

The gestational period exerts considerable influence on the general health of offspring,^75^, ^76^ including obesity.^77^ Studies indicate that gestational weight gain is of relatively small importance relative to maternal BMI.^78^ Indeed, maternal influence on long‐term BMI of offspring appears to be no different from the paternal–offspring BMI association.^79^ This suggests that fetal overnutrition may be of minor importance, especially when compared to maternally transmitted genetic risk.^80^

There may be an important influence of maternal stress during the gestational period,^81^ leading to increased stress vulnerability and obesity in offspring.^82^ Furthermore, children whose mothers have been exposed to considerable stress before and during the gestational period (as determined by loss of close family members during the months before conception and during gestation) have significantly greater risk of becoming overweight both during childhood and at age around 20 years, apparently with stronger effects of the exposure before than during gestation.^83^

### 3.3 Part B: Insecurity and chronic stress

A central idea of our proposed model, largely derived from developmental psychology, is the understanding that children have basic social and emotional needs, such as sensory stimulation, safety, warmth, and emotional bonding, with parents acting as main providers.^15^ Persistent failures to meet such needs increases the risk of insecurity, chronic stress and negative affect (Figure 1).^20^, ^84^, ^85^

Indeed, when comparing normal weight children with those having overweight and obesity, there is a graded increase in psychosocially stressful life events.^86^ The same pattern has also been noted in adults.^27^ Other factors relating to childhood insecurity and a lack of basic needs include having a missing or disinterested parent, and having divorced or single parents.^87^, ^88^ Insecure attachment has been linked with both eating pathology and excessive weight gain in preadolescents.^89^, ^90^ Particularly harmful social exposures are childhood abuse, maltreatment, neglect and trauma,^9^ which strongly promote insecurity and chronic stress, and which are consistently linked with obesity and a plethora of health‐damaging issues.^9^, ^24^, ^25^, ^91^ Furthermore, children, unlike adults, have yet to develop adequate strategies for coping with social adversity, making them more vulnerable to developing insecurity and heightened stress reactivity.^92^, ^93^

### 3.4 Stress‐mediated pathways to obesity

There are several proposed pathways from social adversity to weight gain and obesity, for example via an increase in energy intake resulting in a positive energy balance,^94^, ^95^, ^96^ a so‐called “push” mechanism of energy storage.^97^ There is also a link between poverty and chronic stress with consuming food with a high caloric density,^10^ potentially mediated by the sympathetic nervous system and the hypothalamic–pituitary–adrenal (HPA) axis, which becomes hyperactive when living in stressful circumstances,^91^, ^98^ or through neurobiological adaptations that increase food reward while also reducing inhibition.^99^ However, the direction of association between an excessive energy intake and obesity is not always clear. It remains a distinct possibility that changes in food intake may also be a consequence of a high or increasing body weight (mainly driven by an increase in lean body mass) rather than a cause, a “pull” mechanism, due to the increased energy needs to develop and maintain a larger body mass,^97^ or other mechanisms such as insecurity and chronic stress.^100^, ^101^

Chronic stress and allostatic load, modulated by neuropeptide Y and glucocorticoids, is also associated with important changes in metabolism, such as decreased post‐meal energy expenditure, lower fat oxidation, and increased insulin production.^100^, ^101^, ^102^ Moreover, individuals with obesity tend to preserve more energy in the post‐physical activity phase than normal weight individuals,^103^ suggesting that energy expenditure is negatively affected. Findings such as these indicate that individuals with obesity may have altered their defended body weight set point upward,^104^ presumably regulated in the brain, conceivably through prolonged social adversity, insecurity, chronic stress and negative affect,^18^ or other adverse environmental factors.^105^

Another hormone mediating the effects of social factors on weight gain is oxytocin. During infancy, there is a high degree of cortical reorganization, via oxytocin pathways, from non‐social to social functions.^106^ A central force behind such evolutionary repurposing is the use of social mechanisms to manage stress, particularly in adapting to harsh ecologies and in managing life as part of large social groups.^106^, ^107^ Oxytocin is key in the development of human social behavior, in social stress regulation and in the development of trust.^90^ Among the functions of maternal oxytocin during breastfeeding is infant regulation of HPA activity, contributing to health, cognitive function, and social adaptation.^108^ Positive attachment is associated with higher oxytocin levels and lower HPA responses to stress.^109^ Oxytocin is also implicated in satiety and in ameliorating stressful experiences.^110^

### 3.5 Part C: Obesity and weight stigma

Once a child has developed overweight or obesity, a new and highly debilitating type of social adversity starts to operate in the form of weight stigma (Figure 1). Girls and women are particularly affected,^23^, ^111^ likely due to greater slim‐ideal internalization, concerns about body shape and image, and social pressure to conform to cultural norms.^112^, ^113^

Weight stigma in many ways contributes to a circular causal process, whereby stigma results in greater stress levels, further weight gain, and further stigma in turn.^23^, ^26^, ^114^, ^115^, ^116^ Weight stigma has been found to be associated with an elevated risk of several obesity comorbidities and mortality independent of BMI, highlighting its profound psychological and physiological influence.^23^, ^26^, ^95^, ^114^ An additional consequence of weight stigma is a tendency to promote excessive dieting, eating disorders, mental health problems, loss of lean tissue, and worsening obesity.^23^, ^117^, ^118^, ^119^, ^120^

Crucially, of the many and various stressors that humans encounter, the social and negatively evaluative ones (including rejection) most engage the HPA axis.^121^ Weight stigma therefore adds considerably to the already high social adversity burden experienced by most individuals with obesity^115^ and is pervasive at all levels of society.^23^ If anything, social pressure to be thin has increased across different societies during the last decades.^23^, ^115^ Part of this effect can likely be traced to the cultural shift away from social cohesion toward values of individual responsibility, self‐evaluation, and competitiveness,^122^, ^123^ where failure to conform to body weight norms is generally met with socially punitive actions.^111^, ^113^ This not only promotes further insecurity and stress but also debilitating affective states such as shame, guilt, and lower self‐worth, common in individuals with obesity.^18^

## 4 MODEL BOUNDARIES AND APPLICATIONS

An important task in obesity research is to identify how an increasing number of biological predispositions and environmental exposures might operate together.^124^ The case for such a shift in approach to understanding obesity as involving complex systems, as opposed to the more classical approach of identifying independent risk factors, has been argued persuasively.^125^, ^126^, ^127^ The present model is one such attempt, which forms a subsystem of a more complete system of obesity causation.^124^ The rationale for this approach is to facilitate wider understanding of the multitude of involved social factors, mainly related to adversity and insecurity, and their interconnectedness with human genetics (i.e., the likely modulation by genetic predisposition of prolonged exposure to social adversity to promote obesity development).^128^ However, other potential subsystems of obesity causation also need to be integrated, notably behaviors (diet, sleep, physical activity), health care (access to prevention, medication, etc.), and macro‐environmental factors (policies, economics, social structures, etc.).^97^, ^104^, ^127^, ^129^ Moreover, we also need to identify new and more system‐based areas for prevention that allow for upstream interventions that have multiple impacts to be identified.^125^, ^126^, ^127^, ^130^

Experimental studies show that early childhood investments in deprived areas to enrich the social and educational environment of children results both in short‐term and in long‐term health benefits, including lower rates of obesity in adult years.^131^ An experiment relating to the promotion of greater social capital and cohesion within financially deprived neighborhoods has shown that help with moving from an area with a high level of poverty to one with less poverty results in a reduction in severe obesity (BMI ≥ 35 kg/m^2^) and type 2 diabetes.^132^ There is also the possibility of adding social media networks to prevention initiatives.^133^ Strategies such as these are seemingly much aligned with the abovementioned principles of social contagion pathways.^57^, ^134^

The lack of data from countries outside Europe and North America is of particular concern, because the association between socioeconomic status and obesity in higher income countries tends to be the opposite to lower income countries,^4^ differences possibly explained by the relative food abundancy in different population segments in these countries. Finally, although a few studies are based on quasi‐experimental design,^49^, ^50^ or a truly experimental design,^131^, ^132^ indicating causality, we acknowledge that the majority of studies on the role of social adversity in obesity causation are studies of association, susceptible to well‐known scientific limitations.

## 5 POSITIONING OF THE MODEL WITHIN THE EXISTING LITERATURE

It is much beyond the scope of this paper to review all pertinent theoretical models of obesity development, for example, interactions of social adversity with other subsystems, such as behavior. Alternatively, we provide some positional context of how this model compares to previous efforts within the social domain. The present model is largely an attempt to expand on earlier studies within the specific area of childhood abuse, maltreatment, neglect, and weight stigma.^8^, ^9^, ^23^, ^24^, ^25^ Since these earlier studies, there has been a steady increase in the number of studies linking such social exposures to obesity development, for example, by Schroeder et al and Wiss et al, including a discussion of plausible mechanisms (social disruption, health behaviors, and chronic stress response).^135^, ^136^ The realization that there is a consistent association between childhood abuse and obesity development has spurred an attempt to describe the role of psychological and emotional distress, a common consequence of childhood abuse, in weight gain and obesity.^18^ This previous model does not, however, include any thorough discussion of the tendency of obesity to be transmitted from parents to offspring (particularly the genetic influence). Moreover, there is no description of how social consequences of obesity, mainly weight stigma, appear to be a key part of obesity development,^23^, ^26^, ^115^ fueling the vicious circle both across and within generations, illustrating the changing nature of social adversity throughout the life course. Speaking more conceptually, the proposed model may be considered akin to a biopsychosocial approach to understanding disease etiology, involving a complex sequence of cascading social, psychological, emotional, and physiological events, eventually resulting in an excessive accumulation of adipose tissue,^20^ provided there is a biological foundation for this process. Clearly, the great variation in genetic predisposition to obesity must be integrated in the model, both because of the direct effects on these biological processes and because of the possible modulation of the effects of the exposures.^128^, ^137^

We acknowledge that there is considerable overlap between our proposed model with models such as the Health Capabilities approach by Prah‐Ruger,^127^ that focuses more broadly on health through the combined influence of biology/genetics, intermediate social contexts, public health and health care systems, and the macro social, political, and economic climate. Our proposed model may be viewed as a more in‐depth exploration of the link between biology and the intermediate social environment specifically for obesity while also recognizing that this is done in the context of other important macro and health care influences.^127^

## 6 MODEL‐BASED SUGGESTIONS FOR PREVENTION

Although there are no simple solutions to obesity, we argue that there is nevertheless considerable potential to improve the current situation. This will likely require a concerted systems‐based approach to improve overall social living conditions, especially for families, but also at the level of the individual and communities.^138^, ^139^ Although such initiatives may be laudable, they are unlikely to succeed unless accompanied by many other improvements to reduce the obesogenic nature of many societies now, including changes to food systems, urban planning, cultural and behavioral norms and values, health care provision, and market regulation, among many other contributing factors.^124^, ^140^, ^141^ Concerted policy changes are therefore a likely prerequisite for reaping lasting and meaningful benefits (Table 1). It is also important to recognize that such changes expectedly will have beneficial effects on public health extending much beyond the prevention of obesity, including the prevention of many common chronic diseases.

**TABLE 1:** Model‐derived proposals for mitigating social adversity relevant to obesity prevention at the level of populations, communities, families, and individuals

Level	Factors	Mitigating interventions
Population/country	Poverty and financial insecurity Lack of parental education Inequality Unsafe cities Neoliberal values Pervasive weight stigma	Financial support Expanded social investments Facilitation of higher education Taxation of obesogenic food products Promotion of healthy schools and cities Greater emphasis on prevention in health care Antidiscrimination laws
Local community	Neighborhood deprivation Low social capital Low social cohesion and crime	Neighborhood safety and social enrichment Building community social capital
Family/household	Parents with obesity Financial insecurity Low education Poor family dynamics Housing quality	Financial support for vulnerable families Paid parental leave Maternal and child health
Individual	No breast feeding Neglect Lack of support Lack of social security Food insecurity Mental health problems	Maternal and child/school health Support for education Healthy lifestyle choices Promotion of balanced norms and ideals

In terms of strategies for mitigating social adversity, the natural experiment study by Barcellos et al suggests that additional education levels can be an effective strategy for reducing obesity inequality,^130^ as is reducing financial strain and poverty.^142^ Although politically and culturally challenging, this could potentially facilitate improved overall social living conditions, which in turn can prevent many of the more downstream psychological, emotional, behavioral, and physiological aspects of obesity development, consistent with complexity theory.^126^ Another strategy to improve the social environment is to use participatory action research.^143^ This is a “bottom‐up” strategy where researchers and participants jointly seek to understand history, culture, and local contexts to shape new action‐oriented research on how to improve obesity prevention outcomes.^144^ This could also be tailored more to subgroups of individuals where social adversity is likely to play a greater part than for other subgroups, where other exposures than social adversities may be more harmful.

## 7 SUGGESTIONS FOR FUTURE RESEARCH

To improve understanding of how social factors influence obesity development, we suggest three main areas of future research, which we were not able to incorporate in our review. Firstly, empirical investigations into obesity etiology would benefit from a wider selection of variables to include multiple subsystems of obesity causation. Examples include markers of genetic predisposition and social factors but also diet, nutrition, sleep, physical activity, metabolism, mental health, amongst many others. Given the strong tendency of obesity to transition from parents to offspring, data on parents (BMI, education level, income, etc.) will likely be highly informative. Secondly, there is a need to advance the understanding of the biological mechanisms linking the psychosocial domain to the fat accretion in adipose tissue, which may be facilitated by employment of the various “‐omics” techniques (genomics, metagenomics, epigenomics, metabolomics).^96^, ^128^

Finally, there is also a need to perform a comparative, comprehensive review of theory‐based literature on proposed models of obesity development. A related priority is to assess the positioning and predictive ability of the current model in relation to other obesity development models and to develop methods of testing the validity of the respective models. This would require a separate publication, and therefore beyond the scope of the current paper. We fully recognize that the generation of new models of obesity development pertaining to the social domain is both an iterative and incremental process, where the final test is to empirically assess how such models perform in a real‐life environment, although there are many scientific, practical, political, cultural, and ethical challenges of doing so. Our expectation is that the presented model offers a parsimonious explanation of the observed strong associations of social adversities across and within generations but other model elements will likely be needed to explain the differences over time and between geographical areas in occurrence of obesity.

## 8 CONCLUSION

Social factors (mainly different forms of prolonged social adversity), especially when combined with genetic predisposition, play an important upstream role in obesity development, by creating a cascade of psychological, emotional, behavioral, and physiological processes, such as insecurity, negative emotions, and chronic stress, that are either directly or indirectly involved in weight gain and obesity. Once obesity is manifest, social adversity is further compounded by debilitating weight stigma, fueling a vicious circle of toxic stress and further weight gain. Genetic factors influence both social adversity and physiological pathways to obesity (genetic pleiotropy), greatly contributing to a highly probable transmission of obesity from parents to offspring, but also to obesity development within current generations. Prevention efforts may be best placed in targeting multiple aspects of social adversity in individuals, families, and communities, notably by mitigating financial strain and increasing education levels.

## CONFLICT OF INTEREST

No conflict of interest was declared.

## AUTHOR CONTRIBUTIONS

EH conceived the study, provided critical input, and drafted the majority of the manuscript; PN provided critical input and wrote parts of the manuscript; SU provided critical input and wrote parts of the manuscript; TIAS provided critical input and wrote a large part of the manuscript. All authors reviewed and approved the final version of the manuscript.
